# Efforts to enhance reproducibility in a human performance research project

**DOI:** 10.12688/f1000research.140735.1

**Published:** 2023-11-01

**Authors:** Jeffrey A. Drocco, Kyle Halliday, Benjamin J. Stewart, Sarah H. Sandholtz, Michael D. Morrison, James B. Thissen, Nicholas A. Be, Christopher E. Zwilling, Ramsey R. Wilcox, Steven A. Culpepper, Aron K. Barbey, Crystal J. Jaing

**Affiliations:** 1 Biosciences and Biotechnology Division, Lawrence Livermore National Laboratory, Livermore, California, 94550, USA; 2 Computing Directorate, Lawrence Livermore National Laboratory, Livermore, California, 94550, USA; 3 Beckman Institute for Advanced Science and Technology and Department of Psychology, University of Illinois Urbana-Champaign, Urbana, Illinois, 61801, USA; 4 Department of Statistics, University of Illinois Urbana-Champaign, Champaign, Illinois, 61820, USA

**Keywords:** reproducibility of results, validation studies, evaluation methodology, data quality

## Abstract

**Background:** Ensuring the validity of results from funded programs is a critical concern for agencies that sponsor biological research. In recent years, the open science movement has sought to promote reproducibility by encouraging sharing not only of finished manuscripts but also of data and code supporting their findings. While these innovations have lent support to third-party efforts to replicate calculations underlying key results in the scientific literature, fields of inquiry where privacy considerations or other sensitivities preclude the broad distribution of raw data or analysis may require a more targeted approach to promote the quality of research output.

**Methods:** We describe efforts oriented toward this goal that were implemented in one human performance research program, Measuring Biological Aptitude, organized by the Defense Advanced Research Project Agency's Biological Technologies Office. Our team implemented a four-pronged independent verification and validation (IV&V) strategy including 1) a centralized data storage and exchange platform, 2) quality assurance and quality control (QA/QC) of data collection, 3) test and evaluation of performer models, and 4) an archival software and data repository.

**Results:** Our IV&V plan was carried out with assistance from both the funding agency and participating teams of researchers. QA/QC of data acquisition aided in process improvement and the flagging of experimental errors. Holdout validation set tests provided an independent gauge of model performance.

**Conclusions:** In circumstances that do not support a fully open approach to scientific criticism, standing up independent teams to cross-check and validate the results generated by primary investigators can be an important tool to promote reproducibility of results.

## Introduction

Reproducibility of findings is a fundamental requirement of any scientific research endeavor. Nevertheless, for a variety of reasons, reproducibility remains a challenge in many areas of the life sciences. ^1^ Batch effects, hidden variables, and low signal-to-noise ratios may interfere with researchers’ ability to draw broad conclusions based on small quantities of data. ^2^ Similarly, data snooping may, even unintentionally, lead to the selection of models that have poor generalizability outside an original dataset. ^3^ These difficulties can be exacerbated in exploratory studies that are by design not limited to the consideration of only one or a small number of pre-specified hypotheses, but rather constructed for the purpose of testing a very large number of possible explanatory variables using a statistical approach.

Transparency in data collection and analysis has been suggested as a potential means of bringing to light methodological or other flaws that may impair the reproducibility of results in various areas of biomedical research. For example, authors who distribute notebooks integrating data, code, and text directly enable others to replicate some or all of the analysis supporting their stated conclusions. ^4^ While such measures do not exclude all possible errors that might call into question the reliability of published findings, scientists adhering to these practices considerably reduce the ambiguity associated with the steps in their workflow subsequent to data acquisition. ^5^^,^^6^

Organizations that fund research must take into account these considerations and others in planning new research and development (R&D) programs. The time and money available to obtain answers to the scientific questions of stakeholder interest are generally limited. Reachback tasking that would allow re-analysis of data or models following an original period of performance is not always possible, as studies frequently rely on teams assembled in an *ad hoc* manner to respond to the requirements of a specific project call. Moreover, publication of results is not a guarantee that the artifacts of a research program will be fully preserved. While many journals have adopted standards for sharing of data and code, compliance with these policies is imperfect. ^7^^,^^8^ Indeed, selective publication practices have themselves been implicated as potential sources of bias in the scientific literature. ^9^^,^^10^ Finally, the aspirations of open science may conflict with project constraints when supporting data are not suitable for release into the public domain.

Here we describe the efforts of one research program, Measuring Biological Aptitude (MBA), a four-year effort sponsored by the Biological Technologies Office of the Defense Advanced Research Projects Agency (DARPA), to improve the reproducibility of studies performed with the goal of optimizing human performance in a variety of athletic and cognitive military skills tests. As the MBA program involved data encumbered by restrictions related to personal privacy, medical confidentiality, and national defense, a fully open approach to promoting reproducibility was not practical. Instead, the program sponsored the authors of this manuscript to conduct a comprehensive independent verification and validation (IV&V) program to test and evaluate the results generated by the primary program contractors and modeling teams.

### Independent verification and validation

DARPA defines IV&V as “the verification and validation of a system or software product by an organization that is technically, managerially, and financially independent from the organization responsible for developing the product” (DARPA Instruction 70). In recent years, IV&V has become a key component of various DARPA research programs both inside and outside the life sciences domain. ^11^ In contrast to the standard in open science, which generally relies on the free and voluntary participation of members of the scientific community to verify the results of third party studies, DARPA’s policy suggests that independent efforts to support the integrity of scientific results are of sufficient importance to merit direct funding, using teams selected for their expertise in the relevant technical areas.

**Figure 1.**
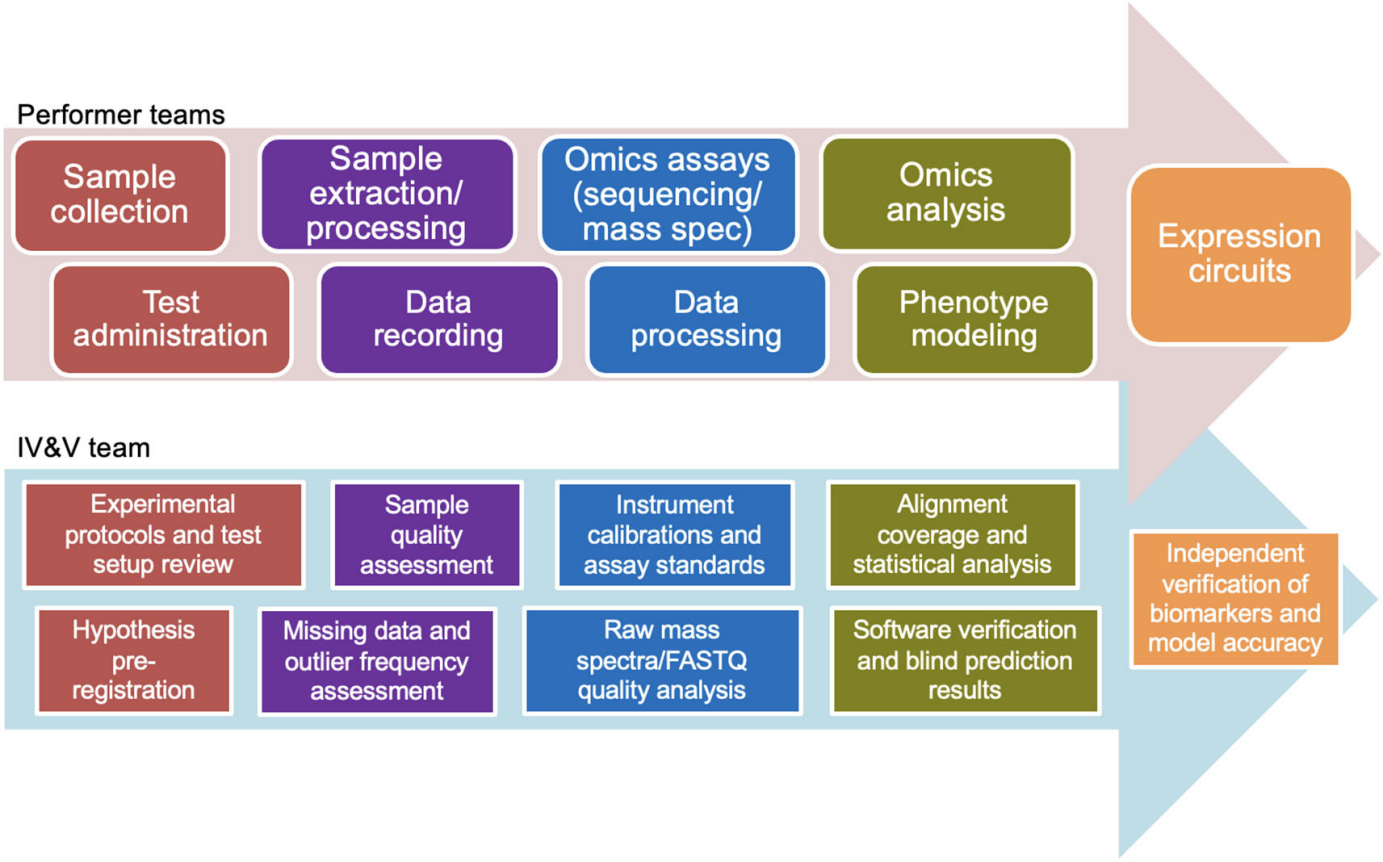
Diagram of the MBA program workflow, denoting the activities of the primary performer teams (top) and corresponding responsibilities of the IV&V team (bottom).

According to the MBA Broad Agency Announcement (BAA), primary performers were charged to “identify, understand, and measure the expression circuits (e.g., genetic, epigenetic, metabolomic, etc.) that shape a warfighter’s cognitive, behavioral, and physical traits, or phenotypes, related to performance across a set of career specializations.” The IV&V team, by contrast, was directed to “verify and validate whether the expression circuits, as measured by the molecular targets identified, directly correlate to dynamic changes in performance traits in the individual and independently confirm…that those circuits correlate to selection success or failure.” From this followed a corresponding but distinct schedule of tasks for each group ( Figure 1).

In 2019, DARPA selected Lawrence Livermore National Laboratory (LLNL) and the University of Illinois Urbana-Champaign (UIUC) to lead the IV&V component of the MBA program. According to the IV&V plan developed by LLNL, the effort comprised four core focus areas: 1) a centralized secure data storage and exchange platform, 2) quality assurance and quality control checklists applied to data acquisition, 3) test and evaluation of performer modeling products, and 4) an archival software repository and data store.

## Methods

### Secure data storage and exchange platform

While the unit costs of bioinformatic data collection have declined in recent years, the acquisition and processing of large-scale omics data remain both financially and computationally expensive. ^12^ For reasons of reliability and cost savings, research sponsors may desire a centralized user facility for data storage and pre-processing, even in projects that involve multiple competing investigators and modeling teams. Moreover, if program managers wish to obtain a comparison of performance across several predictive models, centralized data services may help to maximize the time that data scientists and statisticians are able to devote to model selection while minimizing the risk that ambiguities in outcome labels or other metadata may lead different groups to substantially varying interpretations of the same modeling problem. ^13^^,^^14^

A separate consideration in research involving human subjects involves data security and privacy. In the U.S., federal regulations require institutional review boards (IRBs) to evaluate each proposed project’s provisions for protecting the privacy and confidentiality of human subjects information, regardless of whether a study explicitly plans to include data that is covered by other medical privacy laws. ^15^ In addition, considerations such as the possible re-identification of putatively de-identified health data may warrant additional data protection precautions even when not required by statute or regulation. ^16^

**Figure 2.**
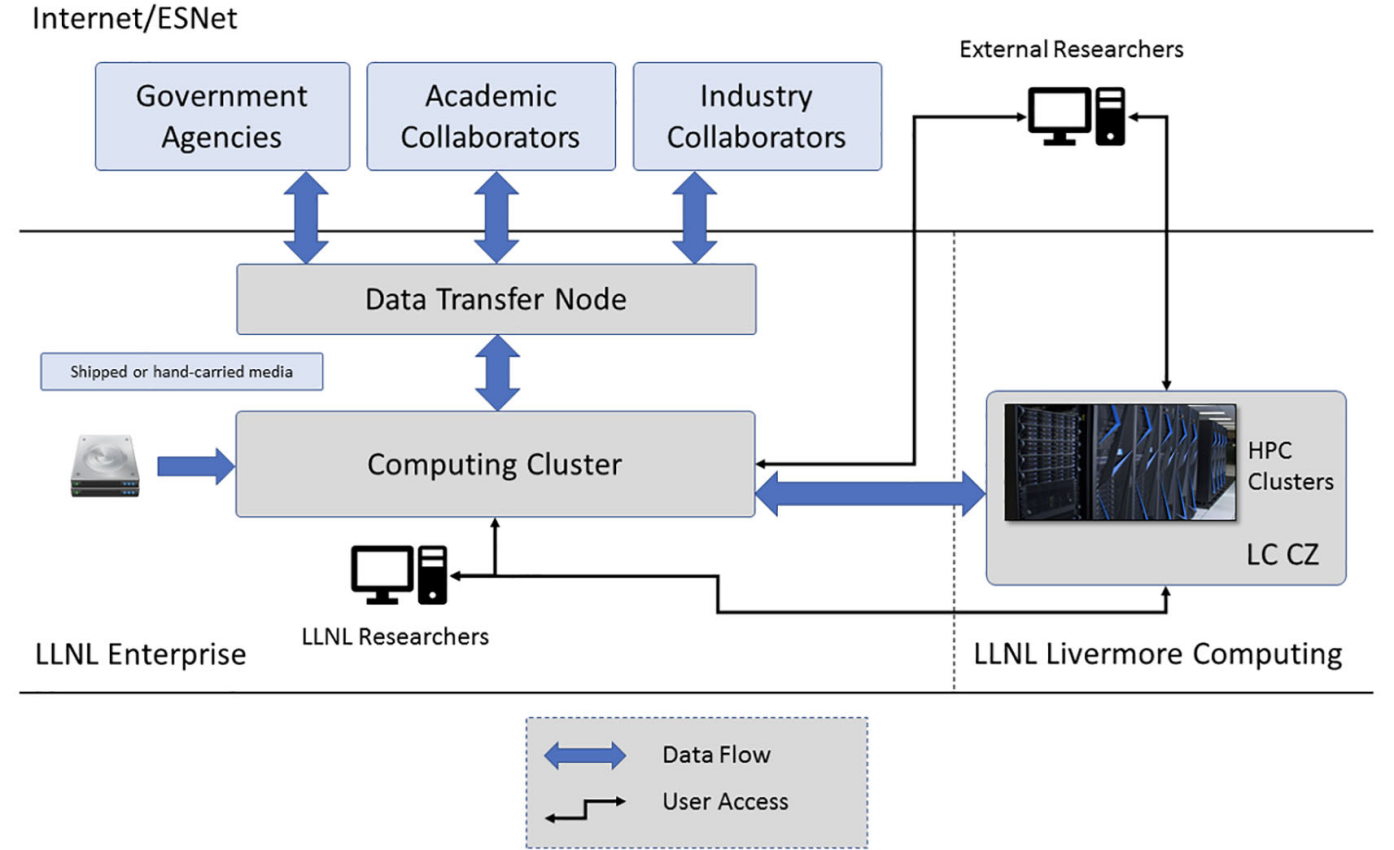
Schematic of the MBA data infrastructure with both a physical enclave (left), including primary compute and data transfer nodes, and an extension to HPC infrastructure (right).

To ensure both data security and data consistency for all teams working on the project, LLNL built a computing enclave for storing and analyzing MBA program data ( Figure 2). Following best practices employed by other centralized biomedical data repositories, the enclave implemented cyber security controls at the FISMA Moderate policy level with enhanced controls from the NIST 800-53 and 800-66 information security guidelines for privacy and HIPAA compliance. ^17^^,^^18^^,^^19^ All accredited users of the enclave were required to complete cyber security training and a human subjects protection course prior to receiving computing accounts. ^20^ Access to the enclave was established through multifactor authentication.

To minimize risks of intentional and/or accidental duplication of human subjects data, the enclave featured a Virtual Network Computing (VNC) portal through which external collaborators could interact with program data. As the enclave excluded other networking protocols for ordinary users, the visual interface allowed modelers to perform analyses in a standard Linux computing environment while imposing a soft barrier against the bulk download of sensitive data. Modelers were allowed to upload new data or software dependencies to the enclave via the data transfer node. However, while outbound transfers of finished analysis were supported via the same pathway, these required the additional step of administrator review and approval.

To permit utilization of high-performance computing (HPC) systems, the LLNL secure enclave was extended to include the Livermore Computing Collaboration Zone (CZ; https://hpc.llnl.gov/hardware/zones-k-enclave). This enabled analysis of multiple omics datasets using leadership-class compute platforms such as Mammoth, a 8,800 core cluster acquired via the National Nuclear Security Administration’s Advanced Simulation and Computing (ASC) Program.

### Quality assurance and quality control of data acquisition

In recent years, various scientific disciplines and consortia have developed minimum standards for the inclusion of data in both centralized repositories and published meta-analyses. ^21^ These guidelines have encompassed a range of data acquisition formats, including genetic, proteomic, and other biochemical data. ^22^^,^^23^ For example, the Human Proteome Organization’s Proteomics Standards Initiative developed the Minimum Information About a Proteomics Experiment (MIAPE) standard for mass spectrometry experiments involving protein and peptide identification. ^24^ Similarly, in the human subjects field, the STrengthening the Reporting of OBservational studies in Epidemiology (STROBE) statement set minimum metadata standards for collection, archiving, and reporting of epidemiological research data. ^25^

Other standards-setting organizations go beyond simple metadata reporting requirements and seek to detail comprehensive processes and systems that can help ensure data quality (quality assurance, or QA) as well as specific tests and benchmarks that can flag errors during and after data collection (quality control, or QC). ^26^ These types of procedural checks have been adopted, for example, by the Metabolomics Quality Assurance and Quality Control Consortium (mQACC) and the Encyclopedia of DNA Elements (ENCODE) Consortium. ^27^^,^^28^ The MBA program used this type of standard as a model for its own QA/QC efforts.

LLNL experimentalists generated a scoring rubric for each of the molecular and omics data collection modalities employed in the various human trials throughout MBA. Example rubrics are shown in Table 1 and Extended Data Tables 1-3. A pass/fail checklist was used to determine whether each dataset met the minimum quality standards for use by the modeling teams. Some criteria involved best practices in sample handling and study design, while others were specific to the instrumentation used and, in general, followed manufacturer recommendations. For some types of data collection, including genome sequencing data, open source tools such as multiQC were utilized as components of the scoring framework. ^29^ Following the IV&V team’s evaluation of each dataset against the rubrics, a scorecard was transmitted to the performer team or subcontractor responsible for the data collection, and the program office was consulted for a final determination on the inclusion of the dataset in the modeling corpus.

**Table 1.** Example QA/QC scoring rubric for immunophenotyping using the CytoFlex-S flow cytometer. Reference ranges are derived from ’CytoFLEX Platform Instructions for Use,’ Beckman Coulter, rev. 12/11/2019. In addition to the pass/fail ranges, some rubrics included a ‘warn’ range for borderline data.

Assay Phase	Specific Metric	Pass Range	Fail Range
Sample Collection	Was sample collected properly (tube type, anticoagulant, etc., per SOP)?	Yes	No
Sample Collection	Was sample properly recorded?	Yes	No
Sample Collection	Was sample stored according to proper procedure for the sample type?	Yes	No
Sample Collection	Is an unbroken chain of custody documented, including dates, times, and locations of all custodian changes?	Yes	No
Sample Collection	Were appropriate transportation conditions used, and were temperatures monitored and recorded?	Yes	No
Sample Prep	Is sufficient sample volume present for processing and analysis?	>10 uL each sample	No
Sample Prep	Were samples thoroughly mixed before loading?	Yes	No
Study Design	Number of Technical Replicates	>1	≤1
Calibration	Most recent quality control and standardization of CytoFlex system (with CytExpert QC)	≤1 day	>1 day
Calibration	Were optical filters verified to match the detector configuration?	Yes	No
Calibration	Were QC fluorospheres adequately mixed?	Yes	No
Calibration	Age of QC fluorosphere preparation	≤5 days	>5 days
Calibration	Was gain set in accordance with manufacturer instructions?	Yes	No
Calibration	Was daily cleaning performed in accordance with instructions?	Yes	No
Experimental	Was threshold adequate to exclude sample debris?	Yes	No
Experimental	Was the detection rate appropriate throughout sampling?	< 10,000 events/second	Other
Experimental	Were positive sample concentrations within acceptable range?	2 ×10 ^4^ − 2 × 10 ^7^ units/mL	Other

Additionally, UIUC statisticians developed separate sets of metrics for the phenotypic and behavioral data collected during the course of the program. These rubrics were crafted to flag outliers and diagnose other potential data quality issues. The analyses encompassed five general domains: cognition, demographics, human performance, personality, and wearable sensors (see Table 2). Some metrics applied to only a single domain, whereas others (e.g., missing data) were relevant for multiple domains. When there is a quality assurance failure, the Potential Issue column of Table 2 provides plausible mechanisms that may underlie the faulty data collection process. The team also developed customized R software scripts that read in the data and automatically generated tables, figures, and reports.

**Table 2.** Example QA/QC scoring criteria for phenotypic data collection. Domains Assessed: Cog=Cognition; Demo=Demographics; HP=Human Performance; Per=Personality; WS=Wearable Sensors.

Domain(s) Assessed	Specific Metric	Response Indicative of Failure	Potential Issue
Cog; Demo; HP; Per; WS	Was there missing data for the participant?	Yes	Data missing randomly or systematically.
Cog; Demo; HP; Per; WS	Were there missing data for > 25% of participants?	Yes	Failure in test administration.
Cog; HP; Per	Was a score an outlier, defined by 1.5 times the interquartile range?	Yes	Visually examine violin plot and magnitude of outlier.
Cog; Per	Were any scores outside the allowed range for the test?	Yes	Data recording error.
HP	Did everyone meet the military-defined threshold?	No	Individuals who failed the threshold are excluded from data analysis.
HP	Was an achieved performance metric beyond human limits?	Yes	Measurement error, data recording, or data transcription issue.
HP	For tests with repeated measures under identical conditions, is the Pearson correlation > 0.75?	No	Measurement error, data recording error, or data transcription issue.
WS	Was data collected?	No	Sensor failure or sensor was not used.
WS	Was data collected for expected duration?	No	Sensor was not always turned on or was not used.
WS	Was data in the expected range?	No	Sensor failure or error in data transcription or processing.
Demo	Is there an entry for each field?	No	Data not entered or deleted.
Demo	Is the field entry an allowed value?	No	Data for fields with unallowed values is unusable.

### Test and evaluation of performer modeling products

The primary deliverable from the MBA IV&V effort was the test and evaluation of performer expression circuit models used to predict achievement on military skills tests. While performers trained statistical models to predict pass/fail outcomes for different candidates on a battery of human performance and cognitive tests, the IV&V team was responsible for certifying to the military cadre that the selected molecular observables were in fact predictive of the chosen outcomes.

Several factors complicated the evaluation of performer models according to these criteria, among them: 1) small sample sizes for program cohorts, 2) the potential for subjective evaluation criteria in certain skills tests, and 3) incomplete coverage in the acquisition of omics data relative to phenotypic data, for which several years of historical data collection were already available.

To mitigate these complications, we implemented a two-pronged model evaluation strategy consisting of both a qualitative component, based on pre-registration of the key mechanistic hypotheses each performer planned to investigate, and a quantitative component, based on an evaluation of the predictions of each performer model against a held-out validation set of true outcome labels.

Hypothesis pre-registration is a technique used in some disciplines to avoid using the same set of data for both hypothesis generation and hypothesis testing. ^30^ Hypotheses proposed by MBA performers at the outset of the modeling effort included a variety of potential biological mechanisms underlying task performance, such as sleep quality, metabolism, muscle tone recovery, and several proposed cognitive/psychological mechanisms. These pre-registration documents were retained by the IV&V team for later determination if the identified predictive biomarkers might plausibly correspond to the pre-specified categories.

For quantitative validation, the IV&V team held back 20-30% of the candidate outcome labels from the primary modeling teams during each year of the MBA program. The outcomes for this validation set were kept fully blinded from performer team members to prevent data snooping. ^31^ Modelers were given all other data from each annual cohort and then asked to submit predictions of the outcomes of the blinded candidates for scoring by the IV&V team. Results were announced at each program review meeting.

### Software repository and data archive

Preserving the ability to apply predictive models to new cohorts of individuals following the conclusion of MBA was a key goal of the program. Given the small sizes of individual cohorts, prospective model testing on future data collection was considered a significant component of the overall validation strategy. Furthermore, the IV&V team desired to ensure that, to the extent possible, the models would be independent of the choice of laboratory for omics data processing to avoid vendor lock-in.

To facilitate a single storage location for program data, LLNL data scientists generated a MariaDB database schema to contain all multi-omic, phenotypic, and outcome data collected over the course of the MBA program. As some omics data was too large to practically store within the database itself, the database contained links to the original and processed data files stored in a master data archive. It also contained metadata to track QA/QC results associated with different datasets, as well as to reconcile individual research subjects with their anonymized identifiers.

To support continued usefulness of the predictive models, the IV&V team requested that performers package their analysis and models for future use as research compendia, according to the method of Marwick et al. ^32^ We chose this format as the R language was the preferred coding environment of the majority of modeling teams. To support long-term portability of the modeling pipelines, containerization of the computing environment using Docker or Singularity was also recommended for each team.

## Results

The primary investigator-led teams funded to perform work for MBA offered a high level of cooperation with our IV&V efforts. We were aided by support for our IV&V plan from the research sponsor, particularly when elements of the plan necessitated extra effort by the performer teams, such as in the case of hypothesis pre-registration or periodic data holdbacks.

Data QA/QC added a modest amount of time between the return of results from experimenters and the availability of processed data for use by modelers. However, on several occasions involving both sequencing and mass spectrometry experiments, issues flagged during the QA/QC process spurred additional consultation with the data collection teams and led to process improvement that was incorporated into subsequent data re-analysis.

Regular holdout validation set tests of performer predictive models provided an unbiased, apples-to-apples comparison of model performance that assisted the sponsor in measuring progress against program goals. Unfortunately, program constraints made it difficult to test counterfactual predictions made by the modelers, i.e., predictions that certain individuals would have progressed further in the selection process than they actually did. As a result, measuring improvement over state-of-the-art in quantities such as recall, as envisioned at the outset of MBA, was not possible. Instead, the IV&V team defaulted to the use of prediction accuracy and F-score as the primary endpoints for model evaluation. ^33^

## Discussion and lessons learned

In recent years, studies have demonstrated that diverse types of omics data are predictive of biological phenotypes supporting human performance characteristics. ^34^ Nevertheless, this field of research comes with a unique set of challenges that separate it from the much larger pool of clinical research seeking to drive progress in the medical domain. “Success” in the human performance context may be a more multifactorial entity than in the medical context, where it may simply entail the cessation of an identified disease process. Additionally, healthy and, in particular, athletically adept individuals may be more reluctant to participate in invasive specimen collection procedures than individuals already engaged with the medical system.

In working with cohorts that significantly depart from broader population baselines, reference data from publicly available databases may turn out to be of lesser value than modelers initially hope. For example, studies of the metabolic impact of various dietary regimens in aging or pre-diabetic populations may not have high transfer value in the warfighter population. To the extent possible, omics data collection for single individuals over long periods of time may mitigate this issue and limit the need for transfer learning from weakly representative populations.

Alternatively, research sponsors may wish to consider funding short-term but larger multiomic studies that are composed of participants more closely representative of the target population. Phenotypic outcomes could be collected passively and unobtrusively using wearables technology. Cadre members could be polled to determine surrogate endpoints, measurable in this more high-throughput context, that they believe most likely related to their more holistic judgments in the selection process of interest. Additionally, if the surrogate endpoint markers are continuously valued, this type of outcome variable may allow for superior statistical power than dichotomized pass/fail outcome labels. ^35^

To participate in blinded prediction contests, modelers may be reluctant to give up scarce training data samples as a validation holdout when the total number of observations in the dataset is small. Statistical techniques that require checking certain prerequisite assumptions, such as the normality of predictor distributions, may become tedious to implement when small amounts of data are released sequentially. The modeler experience might be subjectively improved if there is enough data to constitute multiple test sets, even if some of those are only partially blinded. For example, Kaggle, the competitive data science website, frequently splits datasets into training, “public leaderboard,” and “private leaderboard” components, with the first category being fully accessible to modelers, the second providing a basis for competitors to obtain a preliminary score during the competition, and the final category remaining fully blinded until all models have been submitted. ^36^ Even though the “public leaderboard” data is not truly blinded, since competitors can iteratively query it throughout the model building process, modelers may nevertheless elect to use it judiciously to gauge their performance and to debug basic generalization errors.

Finally, program managers wanting to drive improvements over state-of-the-art outcome prediction, particularly for quantities such as recall, should engage early with program stakeholders to develop means of testing counterfactual predictions made by data scientists. For example, modelers may predict that certain candidates “would have passed” later rounds of a tournament selection process had they been given the opportunity to compete at the higher level. While this information may be of high value from the standpoint of program goals, these predictions are impervious to validation if those individuals are lost to follow-up.

## Conclusions

Community-based efforts to promote reproducibility through open sharing of data and code have played an important role in advancing the methodological rigor of many scientific disciplines. We have demonstrated a paradigm for adapting several aspects of this approach to achieve independent verification and validation of results in the context of a research program where unlimited data exchange is not feasible.

Using holdout prediction tests and hypothesis pre-registration, our team was able to certify that predictive modeling benchmarks were achieved in the absence of data snooping. Additionally, a centralized data infrastructure and integrated QA/QC system promoted data integrity and helped to facilitate the preservation of data and algorithms generated in the course of the project for follow-on research efforts.

While our IV&V strategy was developed for projects at the intersection of human performance and defense, we anticipate that similar protocols may prove useful in other research contexts involving multiomic data analysis and sensitive human subjects data. Though the data and trained models from this project are encumbered by distribution restrictions, other artifacts from the study, such as QA/QC rubrics, have been made available to support future work in this area.

## Data Availability

No data associated with this article. Figshare: Measuring Biological Aptitude Omics QA/QC Rubrics. https://doi.org/10.6084/m9.figshare.23802606.v1. ^37^ This project contains the following extended data:
• ExtendedDataTables.pdf (Sequencing, Proteomics, and Metabolomics QA/QC Scoring Rubrics) Data is available under the terms of the CC-BY 4.0 license.
