# Supplementary material for: Efforts to enhance reproducibility in a human performance research project
Source: F1000Res. 2023 Nov 1;12:1430. [Version 1] doi: 10.12688/f1000research.140735.1 (PMC11406130; doi:10.12688/f1000research.140735.1)
Supplement: Peer review history

# Reviewer report: Reviewer response for version 1

**DOI:** 10.5256/f1000research.154122.r220462

**Published:** 2024-09-16

**Authors:** Elizabeth Dhummakupt

**Affiliations:** 1 U.S. Army DEVCOM Chemical Biological Center, Aberdeen Proving Ground, Maryland, USA

This article discusses a method to assist in reproducibility of data collected and generated by studies in which broad questions are asked and large pools of participants are not always available. Additionally, the methods proposed here are useful when full and open data sharing is not always possible, as in the case with research involving national security implications. 

 The method discussed herein is easily implemented in that adherence to rubrics is required, which can be done without adding software packages or investing in high performance computing.

Is the rationale for developing the new method (or application) clearly explained?

Yes

Is the description of the method technically sound?

Yes

Are the conclusions about the method and its performance adequately supported by the findings presented in the article?

Yes

If any results are presented, are all the source data underlying the results available to ensure full reproducibility?

No source data required

Are sufficient details provided to allow replication of the method development and its use by others?

Yes

Reviewer Expertise:

Multi-omic biomarker discovery - proteins, lipids, metabolites

I confirm that I have read this submission and believe that I have an appropriate level of expertise to confirm that it is of an acceptable scientific standard.

# Reviewer report: Reviewer response for version 1

**DOI:** 10.5256/f1000research.154122.r233981

**Published:** 2024-03-19

**Authors:** Rahuman S. Malik‐Sheriff

**Affiliations:** 1 European Bioinformatics Institute (EMBL-EBI), Cambridge, UK

The authors present a methodology they used to ensure the reproducibility and validation of model predictions in the MBA program, where it was not possible to publicly share the raw data due to privacy concerns or the sensitivity of the information. The authors propose and perform independent verification and validation (IV&V) of markers identified using multi-omics data analysis to predict human performance; using holdout validation sets that are not shared with the project contractors to allow unbiased evaluation of model performance. This approach is quite reasonable, the authors present the limitation in assessing the counterfactual prediction of what would have happened in scenarios where the data was not acquired.

 Overall, it is a very good piece of work, which fairly discusses the merits and limitations of these approaches. Holdout validation seems like an excellent strategy for the validation of models developed by diverse groups using the same set of data.

 Adding some quantitative table or figure showing, how many of the hypotheses/findings were independently verified and validated and how many were reproducible/not reproducible/non-verifiable, could strengthen the manuscript. If possible, some high-level categorization of the models/predictions will provide a bit more context.  

 The IV&V strategy includes implementation of 4 steps: 1) a centralized data storage and exchange platform, 2) quality assurance and quality control (QA/QC) of data collection,

 3) test and evaluation of performer models, and 4) an archival software and data repository. These four steps are elaborately discussed in the manuscript, however, there are a few questions raised below. If the authors find it reasonable, it would be useful to discuss / clarify them in the manuscript.

 It would be worthwhile discussing how the platform developed in step 1 differs from federated learning platforms and existing strategies, including their merits and demerits, if any.

 Baker 2016 reported that over 70% of the researchers failed to reproduce other scientists’ experiments and 50% failed to reproduce their own.  In step 2, concerning QA/QC, is it sufficient to review the experimental protocols and setups? is it not required to independently reproduce a part of these experiments? Can the authors discuss this?

 QA/QC scoring rubric could act as a good guideline for data generation and analysis. However, some of the questions in the QA/QC scoring rubric are a little ambiguous, for example, in question “Was the sample properly recorded?” what does ‘properly’ mean?

 Are questions such as “Were samples thoroughly mixed before loading?” useful? has anyone ever answered no to these questions?

 In step 3, the authors suggest registration of the hypothesis, however, in most cases where multi-omics data is used, it is often data-driven hypothesis generation, can the authors discuss this? How often are the hypotheses registered are successfully verified by both parties?

 Tiwari *et al.* 2020 showed that about half of the computational model cannot be reproduced using the information provided in the manuscript. In step 3 for validation, did LLNL use the same codes originally developed / used by the researchers?  were they verified and validated to ensure that any issues in the code were appropriately addressed?  Any changes to the steps in processing data, including data normalization will impact the prediction, so where any standards and best practice guidelines are recommended to researchers to harmonize the analysis.

 In Step 4, though the data cannot be publicly shared, can some of the software / codes / models? be shared publicly in accordance with FAIR data-sharing principles. It would be critical to ensure these are reusable if the plan is to apply these models on new cohorts.

 As a side note, with some rewriting, the abstract and overall article could be well enhanced to appeal to a broader audience.  Here are some of the points. These are optional recommendations; the authors can consider these and similar others in the article and address them as they feel appropriate.

- In abstract, if “one human performance” is the name of the research program, using punctuation / capitalization appropriately will be helpful, otherwise please rephrase the text accordingly.
-  In abstract: methods could be improved to increase the clarity. The authors could clarify that they present the method they developed and performed a third party independent verification and validation of outcomes of the DRDO-funded projects.
-  In abstract: Holdout validation set, could be clarified.
- Clarifying in the abstract that human performance prediction models based on multi-omics data will give a bit more context. If space allows, it would be useful to mention that performance of war-warriors performances are predicted.
- In general, providing some definition for terms such as ‘data snooping’, and ‘expression circuits’ would be helpful.

Is the rationale for developing the new method (or application) clearly explained?

Partly

Is the description of the method technically sound?

Partly

Are the conclusions about the method and its performance adequately supported by the findings presented in the article?

Partly

If any results are presented, are all the source data underlying the results available to ensure full reproducibility?

No source data required

Are sufficient details provided to allow replication of the method development and its use by others?

Partly

Reviewer Expertise:

I am a computational biologist with interest in standards and reproducibility of computational models.

I confirm that I have read this submission and believe that I have an appropriate level of expertise to confirm that it is of an acceptable scientific standard, however I have significant reservations, as outlined above.

## References

[1] : 1,500 scientists lift the lid on reproducibility. *Nature* .2016;533(7604) : 10.1038/533452a 452-454 10.1038/533452a 27225100

[2] : Reproducibility in systems biology modelling. *Mol Syst Biol* .2021;17(2) : 10.15252/msb.20209982 e9982 10.15252/msb.20209982 33620773 PMC7901289

# Reviewer report: Reviewer response for version 1

**DOI:** 10.5256/f1000research.154122.r233980

**Published:** 2024-03-06

**Authors:** Timothy M. Errington

**Affiliations:** 1 Center for Open Science, Charlottesville, Virginia, USA

The article describes the approach the authors implemented as an independent verification and validation (IV&V) effort within in the DARPA funded Measuring Biological Aptitude (MBA) program. This includes implementing four areas to improve and test the reproducibility of the program performers, particularly within the context of a program where not all data can be openly shared.

 Overall, this is a very nice example of how open science practices can be implemented within a program like this. My suggestions are directed on clarifications and expansions to help broaden the readership of this article.

1. Additional context about how IV&V is typically implemented in related programs and why these specific four features were developed.
2. The benefits (and costs) of implementing open science and reproducibility steps in a program like this. That is, IV&V is running ‘alongside’ the performers. However, outside large programs it is more typical researchers (i.e., performers) disseminate their research and then other researchers (i.e., IV&V) look at the paper and outputs (e.g., data, code).
3. In addition to discussing the steps that were implemented (and expanding on the rational), how did the specific improve the program? That is, in the authors opinions, did the effort to implement these steps increase the overall model evaluations? At the end of the results the pivot the IV&V team took to evaluate performance is indicated, but the impact of these measures is absent – this doesn’t have to be disclosing the specific F-scores, more how the four areas that the IV&V team implemented lead to improvements (however that is defined) of the performers and program at large.
4. I think if possible, reworking Figure 1 to highlight not only the two tracks and primary responsibilities of the performers and IV&V, but how this maps onto a more general workflow of research (particularly at the intersection of human performance and defense) would be helpful. I think the abstraction beyond the specific program would help strengthen the conclusion the authors are making about this approach being useful for other projects.

Minor comments:

1. Was pre-registration implemented by performers and used by the IV&V team (which is how I read it from the text), or was it implemented by the IV&V team (which is what Figure 1 indicates. Related, are any pre-registrations openly available or is this also ‘encumbered by distribution restrictions’?
2. Within the Independent verification and validation section of the Introduction there are extra periods in one of the sentences (“confirm…that those circuits”)

Is the rationale for developing the new method (or application) clearly explained?

Yes

Is the description of the method technically sound?

Yes

Are the conclusions about the method and its performance adequately supported by the findings presented in the article?

Partly

If any results are presented, are all the source data underlying the results available to ensure full reproducibility?

No source data required

Are sufficient details provided to allow replication of the method development and its use by others?

Yes

Reviewer Expertise:

open science, reproducibility, metascience, molecular and cell biology, preregistration

I confirm that I have read this submission and believe that I have an appropriate level of expertise to confirm that it is of an acceptable scientific standard, however I have significant reservations, as outlined above.

# Reviewer report: Reviewer response for version 1

**DOI:** 10.5256/f1000research.154122.r233971

**Published:** 2024-01-19

**Authors:** Sheeba Samuel

**Affiliations:** 1 Friedrich Schiller University Jena (Ringgold ID: 9378), Jena, Thuringia, Germany

In the context of human performance research, the authors present their study focusing on the Measuring Biological Aptitude (MBA) research program, organized by the Defense Advanced Research Project Agency's (DARPA) Biological Technologies Office. To address reproducibility, the authors implemented a four-pronged independent verification and validation (IV&V) strategy, including centralized data storage, quality assurance and control of data collection, evaluation of performer models, and an archival software and data repository. The IV&V plan, carried out with support from the funding agency and research teams, involved processes like quality control, error flagging, and holdout validation set tests. The paper addresses a topic which underscores the significance of reproducibility, particularly in situations where a fully open approach is unfeasible. In such scenarios, the paper advocates for the establishment of independent teams to cross-check and validate results as a pivotal step in promoting research reproducibility.

 To offer the necessary motivation in the introduction, the paper could explore the challenges associated with ensuring reproducibility in sensitive fields where privacy concerns limit the open sharing of raw data. It would be beneficial to highlight the obstacles to reproducibility, particularly when faced with limitations related to personal privacy, medical confidentiality, and national defense. The authors could also discuss how other state-of-the-art approaches address these challenges and explore the potential applicability of this approach in similar programs within and outside DARPA research initiatives. Additionally, the authors could provide explanations for introduced terms which are not known to every readers such as data snooping, Reachback tasking, performer expression circuit models, and Holdout validation within the manuscript. Before going into the IV&V methods, a brief introduction and background on the complete test program, and data acquisition w.r.t the large scale omics data would enhance the reader's understanding. The discussion section introduces various new terms and statements that were not previously mentioned, creating a disconnect from the previous sections. It is imperative to establish a clear connection between the concept of reproducibility mentioned in the introduction section and the insights gained in the discussion section. This connection should explicitly define reproducibility in the given context and elaborate on how it is achieved, especially considering the constraint of not being able to share the data online. The paper could also discuss the limitations and the problems occurred in their context, along with proposing potential enhancements that the funding agency could implement to foster reproducibility in similar situations.

Is the rationale for developing the new method (or application) clearly explained?

Yes

Is the description of the method technically sound?

Partly

Are the conclusions about the method and its performance adequately supported by the findings presented in the article?

Partly

If any results are presented, are all the source data underlying the results available to ensure full reproducibility?

No source data required

Are sufficient details provided to allow replication of the method development and its use by others?

Partly

Reviewer Expertise:

My research focuses on reproducibility, FAIR principles, knowledge engineering, provenance, and research data management.

I confirm that I have read this submission and believe that I have an appropriate level of expertise to confirm that it is of an acceptable scientific standard, however I have significant reservations, as outlined above.

# Reviewer report: Reviewer response for version 1

**DOI:** 10.5256/f1000research.154122.r220464

**Published:** 2023-11-22

**Authors:** Michael Fitzsimons

**Affiliations:** 1 The University of Chicago, Chicago, Illinois, USA

The authors share their approach to performing an Independent Verification and Validation (IV&V) process for the Measuring Biological Aptitude (MBA) project, which is funded by the Biological Technologies Office of the Defense Advanced Research Projects Agency (DARPA). They discuss IV&V as a general use case for supporting research reproducibility in a field where data cannot be directly shared. They also provide the specifics of their implementation including QA/QC and qualitative and quantitive evaluation of model performance. I found the article interesting and informative, but I think it is lacking some context that would help readers to understand more about IV&V. In particular, I would like to see considerably more detail about how their approach differs from or improves up other IV&V examples. A cursory review of the literature shows many papers on the topic and it would be helpful to understand how the authors' approach differs and compares.

 I would also love to see any more details about their actual model evaluation techniques. I presume the sponsors constrains what they are allowed to share, but at present it is somewhat difficult to assess the rigor of their verification and validation process.

 Additional small and targeted comments are included below:

**Introduction**

 Data snooping - I don't think this term is understood by all. It might be helpful to add another sentence or phrase about data snooping.

 Reachback tasking is not a standard term as far as I know and should be modified or explained.

 Independent verification and validation (IV&V) - I would like to see a lot more discussion of what this typically looks like and how your implementation compares.  A casual review of the literature shows lots of citations about "independent verification and validation (IV&V)" - how does your paper add to this literature?. I cannot tell if your implementation is good, bad, or typical. Given we cannot see any actual modeling results (I assume this is a restriction by the sponsor), I think it is important to include a broader comparison to the existing IV&V literature.

**Methods**

 Standards discussion is good.

 Could you really ascertain the answer to some of the QA/QC questions such as "Were collection tubes labeled with unique, traceable codes?" and "Were samples thoroughly mixed before loading?"

 Can you include any more details about the actual models or their evaluation? This is for the quantitative evaluation. Or maybe at least say explicitly what the limitations of what you are allowed to provide.

**Results**

 Are there any results, or at least the format for results, that you can share?  

**Other suggestions**

 Mention privacy preserving federated learning as another option for incorporating data that cannot be shared directly.

Is the rationale for developing the new method (or application) clearly explained?

Yes

Is the description of the method technically sound?

Yes

Are the conclusions about the method and its performance adequately supported by the findings presented in the article?

Partly

If any results are presented, are all the source data underlying the results available to ensure full reproducibility?

No source data required

Are sufficient details provided to allow replication of the method development and its use by others?

Yes

Reviewer Expertise:

Genomics, data sharing solutions, software

I confirm that I have read this submission and believe that I have an appropriate level of expertise to confirm that it is of an acceptable scientific standard, however I have significant reservations, as outlined above.
